# Supplementary material for: Application of plasma metabolome for monitoring the effect of rivaroxaban in patients with nonvalvular atrial fibrillation
Source: PeerJ. 2022 Aug 9;10:e13853. doi: 10.7717/peerj.13853 (PMC9373988; doi:10.7717/peerj.13853)
Supplement: File S1 [file peerj-10-13853-s002.docx]

Supplemental Methods

*Sample Enrollment*

The enrolled NVAF patients were evaluated by cardiologists in Beijing Hospital as suitable for treatment with rivaroxaban and did not have rheumatic valvular disease or prosthetic heart valves (Camm et al. 2012). Patients with atrial fibrillation that was reduced by some reversible factors, such as perioperative onset and hyperthyroidism, were also excluded.

The study contained two parts, one is the plasma metabolome analysis of control group and patients with atrial fibrillation (before rivaroxaban intake); another is the plasma metabolome analysis of patients with atrial fibrillation before and after rivaroxaban intake. The plasma was collected from controls, patients with atrial fibrillation (before rivaroxaban intake) and the same patient with atrial fibrillation (after 3h rivaroxaban intake). The sample of the patients with atrial fibrillation (before rivaroxaban intake) was collected before rivaroxaban intake. According to some pharmacokinetic studies of rivaroxaban, rivaroxaban reached a steady state concentration at day 4–5 after administration (Freyburger et al. 2015; Mani et al. 2011). Then the NVAF patients take rivaroxaban once orally at 8am for at least four consecutive days. In the fifth day when rivaroxaban had reached a steady state concentration, the patients take the drug at 8am and the plasma samples were collected at 11am.

*Sample Preparation*

First, LC–MS/MS-based plasma differential metabolome analysis was performed in 75 NVAF patients (before and 3 h after rivaroxaban intake) and 50 controls to discover candidate biomarkers for NVAF (before rivaroxaban intake vs. control), as well as for laboratory monitoring of rivaroxaban (before vs. 3 h after rivaroxaban intake). Furthermore, the differential metabolites between the two groups were further externally validated by using an independent batch of the other age and gender-matched 75 NVAF patients and 50 healthy controls.

The quality control (QC) sample was a pooled plasma sample prepared by mixing aliquots of two hundred samples across different groups. The samples were randomly selected from NVAF patients and control groups. To evaluate the stability and repeatability of the strategy, the QC sample was analyzed every ten samples throughout the analytical run.

*LC-MS/MS Analysis*

Each sample was analyzed on an HSS C18 column (3.0 × 100 mm, 1.7 µm) (Waters, Milford, MA, USA) by reversed-phase separation. The mobile phase A was 0.1% formic acid in H_2_O and mobile phase B was acetonitrile. The gradient was set as follows: 0–1 min, 2% solvent B; 1–3min, 2–55% solvent B; 3–8 min, 55-100% solvent B; 8-13 min, 100% solvent B; 13-13.1 min, 100-2% solvent B; 13.1-18 min, 2% solvent B. The flow rate was set as 0.5 mL/min. The column temperature was set at 50 °C. The eluted fractions were then analyzed with a Waters ACQUITY H-class liquid chromatograph coupled with an LTQ-Orbitrap Velos mass spectrometer (Thermo Fisher Scientific, MA, USA).

*Quantitative dot blot analysis of metabolites*

We used block randomization method to spilt samples into experimental and validation sets. It is a commonly used method for randomization to prevent potential selection bias (Lim & In 2019; Suresh 2011). We set blocks for randomization and balance the number of subjects in each block. Firstly, samples of each condition (control and nonvalvular atrial fibrillation) were sorted by age and sex. Then the samples were numbered sequentially into 20 blocks. A random number between 0 to 1 were generated for each sample using RAND function in excel. Finally, the samples in each block were sorted by the random number, and sequentially divided into experimental and validation sets.

Reference

Camm AJ, Lip GY, De Caterina R, Savelieva I, Atar D, Hohnloser SH, Hindricks G, Kirchhof P, and Guidelines ESCCfP. 2012. 2012 focused update of the ESC Guidelines for the management of atrial fibrillation: an update of the 2010 ESC Guidelines for the management of atrial fibrillation. Developed with the special contribution of the European Heart Rhythm Association. *Eur Heart J* 33:2719-2747. 10.1093/eurheartj/ehs253

Freyburger G, Macouillard G, Khennoufa K, Labrouche S, Molimard M, and Sztark F. 2015. Rivaroxaban and apixaban in orthopaedics: is there a difference in their plasma concentrations and anticoagulant effects? *Blood Coagul Fibrinolysis* 26:925-933. 10.1097/MBC.0000000000000371

Lim CY, and In J. 2019. Randomization in clinical studies. *Korean J Anesthesiol* 72:221-232. 10.4097/kja.19049

Mani H, Hesse C, Stratmann G, and Lindhoff-Last E. 2011. Rivaroxaban differentially influences ex vivo global coagulation assays based on the administration time. *Thromb Haemost* 106:156-164. 10.1160/TH10-10-0667

Suresh K. 2011. An overview of randomization techniques: An unbiased assessment of outcome in clinical research. *J Hum Reprod Sci* 4:8-11. 10.4103/0974-1208.82352

Supplemental Figure 1. Assessment of QC samples. A). Trend plot showing the variation of t [1] over all QC samples on plasma metabolome. X axis numbers represented sample number; Y axis was arbitrary (3 s.d.); B). PC1 versus PC2 of test samples and QC on plasma metabolomics.


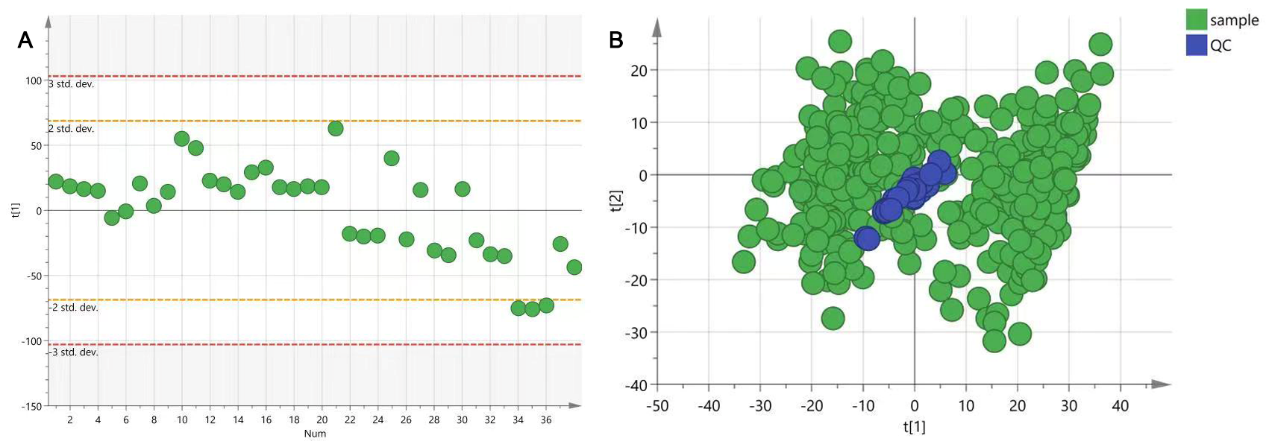


Supplemental Figure 2. The HPLC files of three representative metabolites (avocadene, PE and prenyl glucoside) identified in samples.

1. Avocadene m/z 304.2835,


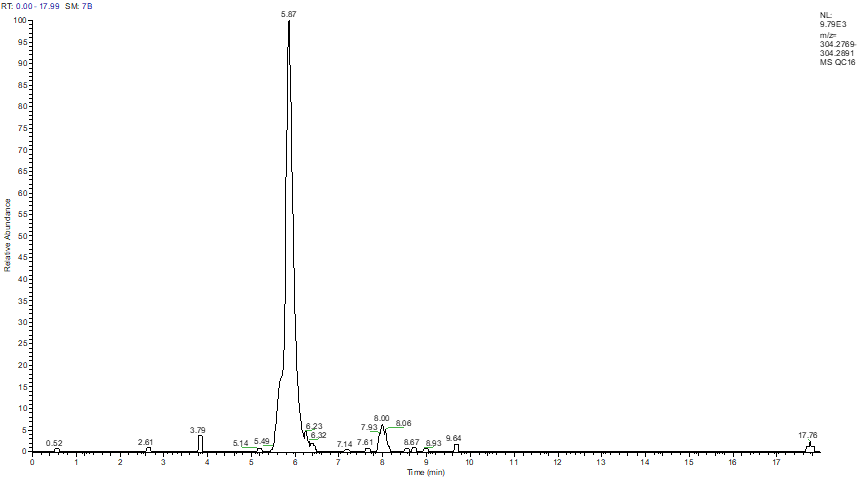


1. PE m/z 780.4558

1. prenyl glucoside m/z 231.1195
